# Antioxidative protection of dietary bilberry, chokeberry and *Lactobacillus plantarum *HEAL19 in mice subjected to intestinal oxidative stress by ischemia-reperfusion

**DOI:** 10.1186/1472-6882-11-8

**Published:** 2011-01-27

**Authors:** Maja Jakesevic, Kjersti Aaby, Grethe-Iren A Borge, Bengt Jeppsson, Siv Ahrné, Göran Molin

**Affiliations:** 1 Food Hygiene, Department of Food Technology, Engineering and Nutrition, Lund University, P.O. Box 124, SE-221 00 Lund, Sweden; 2 Nofima Mat AS, Osloveien 1, N-1430 Aas, Norway; 3 Department of Clinical Sciences, Malmö University Hospital, Lund University, Malmö, Sweden

## Abstract

**Background:**

Ischemia-reperfusion (I/R) in the intestines is an inflammatory condition which activates leukocytes and reactive oxygen species (ROS) and leads to lipid peroxidation and DNA damage. Bilberry and chokeberry fruits are rich sources of polyphenols which may act as antioxidants and prevent lipid peroxidation. Lactic acid bacteria (LAB) may improve microbial status in the intestines and increase the metabolic activity towards polyphenolic degradation. The aim of the study was to clarify antioxidative effects of bilberry and chokeberry fruits alone and with addition of a LAB-strain, *Lactobacillus plantarum *HEAL19, in an I/R-model in mice.

**Methods:**

Male BALB/cJ mice were fed the experimental diets for 10 days. Diets consisted of standard chow supplemented with either bilberry (*Vaccinium myrtillus*) or chokeberry (*Aronia × prunifolia*) powder alone or in combination with the LAB-strain *Lactobacillus plantarum *HEAL19. I/R-injury was induced by holding superior mesenteric artery clamped for 30 minutes followed by reperfusion for 240 minutes. Thereafter, colonic and caecal tissues and contents were collected. Malondialdehyde (MDA) was used as indicator of lipid peroxidation and was measured by a calorimetric assay, lactobacilli were cultured on Rogosa agar plates and *Enterobacteriaceae *on VRBG agar plates, anthocyanins and phenolic acids were analysed by HPLC-DAD-ESI-MSn.

**Results:**

MDA was significantly decreased in the colon of groups fed bilberry alone (p = 0.030) and in combination with *L. plantarum *HEAL19 (p = 0.021) compared to the IR-control but not in chokeberry-fed groups. Supplementation with bilberry or chokeberry alone reduced the total number of lactobacilli on the mucosa. Higher concentrations of anthocyanins were found in the colon than in the caecum content of mice. A more varied composition of different anthocyanins was also observed in the colon content compared to the caecum of bilberry-fed mice. Phenolic acids formed by microbial degradation of the dietary polyphenols in the gut could be detected. More phenolic metabolites were found in the intestines of bilberry-fed mice than in the chokeberry-fed ones.

**Conclusions:**

Bilberry alone and in combination with *L. plantarum *HEAL19 exerts a better protection against lipid peroxidation than chokeberry. These dietary supplements may be used to prevent or suppress oxidative stress.

## Background

Ischemia-reperfusion (I/R) injury occurs when the blood supply returns (reperfusion) to a tissue that temporarily has been deprived of blood supply (ischemia) and triggers an intense inflammatory response [1]. If severe enough, this inflammatory response may result not only in local but also in distant organ injury. I/R causes high morbidity and mortality in both surgical and trauma patients and is associated with organ transplantation, strangulated bowel, vascular surgery and shock [2]. Oxidative stress mediators such as activated polymorphonuclear leukocytes (PMNs) and reactive oxygen species (ROS), which cause lipid peroxidation and protein oxidation, are suggested to play a crucial role in I/R damage [1,3]. Among the internal organs, the intestine is probably the most sensitive to I/R injury [4,5]. A consequence of gastrointestinal I/R is the loss of intestinal mucosal barrier function which results in translocation of bacteria and endotoxin from the gut [2,3].

Antioxidant therapy has been suggested to be efficient in preventing or attenuating oxidative stress and hence I/R injury. The protective effects of the antioxidants in the diet have been attributed to vitamins C and E, carotenoids and plant polyphenols [4-8]. Berries are rich in phenolic compounds, such as flavonoids and phenolic acids, which exhibit a wide range of biological effects, including antioxidant, anti-inflammatory, anticarcinogenic and antimicrobial properties [9]. Bilberries and chokeberries are especially rich sources of flavonoids, mostly anthocyanins which besides being pigments are highly efficient antioxidants [10,11]. Since polyphenols are poorly absorbed from small intestine they are metabolised by colonic microflora into various phenolic and aromatic acids such as derivates of phenylpropionic, phenylacetic and benzoic acids with different hydroxylation patterns [12,13]. Those microflora metabolites of polyphenols are better absorbed in the intestine and may contribute significantly to antioxidant and other physiological effects in the GIT and other tissues [14,15].

The intestinal microflora exerts an impact on the nutritional and health status of the host via modulation of the immune and metabolic functions and provides additional enzymatic activities involved in the transformation of dietary compounds [16]. Inflammation and infection are accompanied by imbalance in the intestinal microflora. I/R in the intestines is an inflammatory condition and, as previously mentioned, causes mucosal barrier dysfunction and translocation of pathogenic bacteria. Probiotics may halt the vicious circle of inflammation by promoting the normalisation of intestinal microflora and exclusion of pathogens, decreasing intestinal permeability, improving the intestine's immunological barrier functions and alleviating the intestinal inflammatory response [17]. The term probiotics is used for live bacteria which have beneficial effects on human health [17]. Most probiotic strains belong to *Bifidobacterium *or lactic acid bacteria (LAB), especially of the genus *Lactobacillus*. *Lactobacillus plantarum *strains produce tannase (the enzyme tannin acylhydrolase) which breaks the galloyl ester bonds of hydrolysable tannins thereby inhibiting their protein-binding properties [18]. Hydrolyzable tannins, such as gallotannin and ellagitannin, are widely distributed in the plants and readily bind with proteins reducing the nutritional value of the foods [18].

The aim of the present study was to clarify the antioxidative effects of the polyphenol-rich bilberry and chokeberry fruits on oxidative stress in mice caused by intestinal ischemia-reperfusion. The combination of the different berries with a LAB-strain of *L. plantarum *HEAL19 was tested in an attempt to enhance the antioxidative effects. Anthocyanins and phenolic acids originating from the berries and from the microbial catabolism of the dietary phenolics were recorded in the caecum and colon.

## Methods

### Chemicals

Di-potassium hydrogen phosphate (K_2_HPO_4_), potassium dihydrogen phosphate (KH_2_PO_4_), tri-sodium citrate dehydrate (C_6_H_5_Na_3_O_7_*2H_2_O), magnesium sulfate heptahydrate (MgSO_4_*7H_2_O), sodium chloride (NaCl), Tween 80, L-cysteine monochloride monohydrate, violet red-bile-glucose agar (VRBG) and formic acid were purchased from Merck KGaA (Darmstadt, Germany). Glycerol was obtained from VWR international (Braire, France) and bacteriological peptone from Unipath LTD (Basingstoke, Hampshire, England). Rogosa agar was purchased from Oxoid LTD (Basingstoke, Hampshire, England). 3-*O*-*β*-glucopyranosides (glucosides) of -delphinidin, -cyanidin, and -petunidin, were purchased from Polyphenols Laboratories AS (Sandnes, Norway). 3,4-dihydroxybenzoic acid (protocatechuic acid), 3,4-dimethoxybenzoic acid (vanillic acid), and 3,4-dihydroxycinnamic acid (caffeic acid) were purchased from Fluka Chemie GmbH (Buchs, Switzerland). Phenylacetic acid, 4-hydroxyphenylacetic acid, 3,4-dihydroxyphenylacetic acid and 3-(4-hydroxyphenyl)propionic acid were purchased from Sigma-Aldrich Chemie GmbH (Steinheim, Germany). 3,4,5-trihydroxybenzoic acid (gallic acid), 3-methoxy-4-hydroxycinnamic acid (ferulic acid), (+)-catechin, and (-)-epicatechin were purchased from Sigma Chemical Co. (St. Louis, MO). 3-phenylpropionic acid was provided from SAFC Supply Solutions (St. Louis, MO). All solvents were of HPLC grade and water was of Milli-Q quality (Millipore Corp., Bedford, MA).

### Animals

Male BALB/cJ mice (Taconic, 8680 Ry, Denmark), weighting approximately 20 g were kept under standard laboratory conditions with a controlled 12-hours light/dark cycle. Animals were acclimatized 1 week before use and had free access to standard animal chow (R3; Lactamin, Stockholm, Sweden) and tap water. The study was approved by the Ethical committee for animal experimentation at Lund University.

### Experimental diets

Each animal was placed in its own cage with a food dish. After 7 days of acclimatisation, animals were fed experimental diets for 10 days. Animals had free access to the diets that were prepared every day and were given to the mice during the dark cycle when they were the most active. Experimental diets consisted of standard chow supplemented with a *Lactobacillus plantarum *HEAL19 strain of human origin, and either bilberry (*Vaccinium myrtillus*) or chokeberry (*Aronia × prunifolia*) grown at Balsgård (Sweden). Berries had been picked, freeze-dried and grounded. The daily dose of bilberry and chokeberry powder, respectively, was 1.6 g/mouse. In order to provide each animal with the same energy amount, I/R control group, sham group and *L. plantarum *HEAL19 group were given 8.7 g of standard chow per mouse and day while berry-groups were given 7.4 g chow per mouse and day. The animal chow R3 was dissolved in water to get softer consistency prior to the addition of the different supplements. *L. plantarum *HEAL19 was mixed in a freezing media (0.85 g/L di-potassium hydrogen phosphate, 0.2 g/L potassium dihydrogen phosphate, 0.6 g/L tri-sodium citrate dihydrate, 0.25 g/L magnesium sulfate heptahydrate, 121 mL 99.5% glycerol, 879 mL distilled water). Dose per day and cage was around 1*10^8 ^cfu. Groups without bacterial supplementation were compensated by adding the same amount of pure freezing media.

### Experimental groups

Animals were randomly divided into the following 7 groups with 9 mice in each group: I/R control group, fed soft standard chow; Sham group, fed soft standard chow; B group, fed standard chow supplemented with bilberries (*V. myrtillus*); B+LplH19, fed standard chow supplemented with bilberries and *L. plantarum *HEAL19 strain (LplH19); Ar group, fed standard chow supplemented with chokeberries (*Aronia × prunifolia*); Ar+LplH19 group, fed standard chow supplemented with chokeberries and *L. plantarum *HEAL19 strain (LplH19); LplH19 group, fed standard chow supplemented with *L. plantarum *HEAL19 strain. All groups, except Sham-group, were subjected to the intestinal ischemia-reperfusion.

### Intestinal ischemia-reperfusion procedure

The mice were anesthetized with 7.5 mg Ketamine (Ketalar 50 mg/mL [Pfizer, UK]) and 2.5 mg Xylazine (Narcoxyl 20 mg/mL [Veterinaria AG, Schweiz]) per 100 g body weight by intraperitoneal injection. The animal was placed on a 37°C warming pad for maintenance of body temperature. A midline abdominal incision was performed and abdominal contents were deflected to the left side. The superior mesenteric artery (SMA) was identified and occluded with a vessel clamp to obtain ischemia of the small intestine and colon and the bowel was returned to the abdominal cavity. Ischemia was confirmed when the intestines became pale. The peritoneal cavity was filled with 1 mL Dulbecco's phosphate buffered saline (PBS) for fluid resuscitation. After 30 minutes of ischemia, the clamp was removed, which resulted in immediate reperfusion. Reperfusion was confirmed with the restoration of colour. The abdomen was closed using a running Vicryl 4-0 suture (Johnson & Johnson, USA). After 240 minutes of reperfusion, the animal was anaesthetized again, sampled and sacrificed. The Sham group was subjected to the surgical procedure described above but without clamping of the superior mesenteric artery. Caecal and colonic contents were used for analyses of polyphenols. Caecal and colonic tissues were rinsed in ice-cold Dulbecco's PBS and used for determination of lipid peroxidation (MDA). The part of ascending colon tissue used for viable counts was placed in 3 mL of freezing media (0.85 g/L di-potassium hydrogen phosphate, 0.2 g/L potassium dihydrogen phosphate, 0.6 g/L tri-sodium citrate dihydrate, 0.25 g/L magnesium sulfate heptahydrate, 121 mL 99.5% glycerol, 879 mL distilled water). All samples were weighted, frozen in liquid nitrogen and stored at -80°C until analysis. Surgery was performed with attention to sterile technique.

### Viable count

Colonic mucosal tissue samples were sonicated for 5 minutes and vortexed for 2 minutes. One mL of the sample was mixed with 9 mL dilution liquid (8.5 g/L sodium chloride, 1 g/L bacteriological peptone, 1 g/L Tween 80 and 0.2 g/L L-cysteine monochloride monohydrate) and serially diluted. After dilution, 0.1 mL of the samples from appropriate dilutions were spread with glass beads (5 mm diameter) on Rogosa agar plates and anaerobically incubated (Gas Pack System, Becton Dickenson Microbiology Systems, Cockeynsville, MD, USA) for 72 h at 37°C (lactobacilli count) and violet red-bile-glucose agar plates (VRBG) aerobically incubated for 24 h at 37°C (*Enterobacteriaceae *count).

### Malondialdehyde (MDA)

Malondialdehyde (MDA) is used as an indicator of lipid peroxidation. MDA-586™ (Oxis International Inc. Portland, Oregon, USA), a colorimetric assay, was used to determine MDA in collected colonic and caecal tissues. Samples were homogenised in 1 mL cold Dulbecco's phosphate buffered saline (PBS) and 10 μL butylated hydroxytoluene (5 mM). After homogenisation samples were centrifuged at 4000 *g *for 10 minutes at 4°C and an aliquot of 0.2 mL of the supernatants were mixed with 10 μL probucol and 640 μL of diluted N-methyl-2-phenylindol. Concentrated hydrochloric acid (150 μL of 12 M) was added to the samples before incubation in a water bath at 45°C for 60 minutes. The samples were then centrifuged at 10 000 *g *for 10 minutes at 4°C, the supernatant transferred to a cuvette, and the absorbance was measured at 586 nm. Since MDA is not stable, tetramethoxy-propane (TMOP) was used as a MDA standard. During the acid incubation step at 45°C the TMOP is hydrolyzed and generates MDA. Unit for expression of MDA is nmol/g tissue.

### Extraction of phenolic compounds

For extraction, 1.5 mL of 80% methanol in water acidified with 1% formic acid was added to approximately 0.2 g of frozen samples of caecum and colon contents. Samples were homogenized with a Polytron Homogenizer PT3100 (Kinematica AG, Littau, Switzerland) for 20 s and sonicated for 5 min in an ultrasound bath at 4°C. After centrifugation at 1300 *g *for 10 min at 4°C the supernatant was collected and kept on ice. Pellet was re-extracted with 1 mL of 80% methanol in water acidified with 1% formic acid. Supernatants were pooled and methanol was removed under nitrogen gas. The weight of the extract was accurately recorded. The weights of the extracts were made up to two times the sample weight by acidified milli pore water. The extracts were filtered through a Millex HA 0.45 μm filter (Millipore Corp., Cork, Ireland) before HPLC-DAD-ESI-MS^n ^analysis.

### HPLC-DAD-ESI-MSn analysis of anthocyanins

The anthocyanins in extracts of colonic and caecal contents were analysed on an Agilent 1100 Series HPLC system (Agilent Technologies, Waldbronn, Germany) equipped with an autosampler cooled to 6°C, a DAD (190-600 nm), and an MSD XCT ion trap mass spectrometer fitted with an ESI interface. The compounds were separated on a Betasil C18-column (250 mm × 2.1 mm i.d., 5 μm particles) equipped with a 5 μm C18 guard column (4.0 mm × 2.1 mm i.d.), both from Thermo Hypersil-Keystone (Bellefonte, PA). The separation was executed with mobile phases consisting of A; formic acid/water (5/95, v/v) and B; formic acid/acetonitrile (5/95, v/v) with the following gradient elution: 0-2 min 10% B, 2-17 min 10-20% B, 17-21 min 20-60% B, 21-25 min 60% B, 25-27 min 60-10% B. The column was allowed to equilibrate for 5 min between injections (10 μL). The column temperature was held at 40°C and the solvent flow rate was 0.25 mL/min.

After UV-vis detection the effluent was introduced directly, without splitting, to the ESI interface where ionization in positive mode was performed. The nebulizer pressure was 40 psi; dry gas flow, 10 L/min; dry temperature 350°C; and capillary voltage 3.5 kV. Ions with *m/z *100 to 2000 were measured, with a scan speed of 27000 amu/s. Fragmentations (MS^2-3^) were carried out in the automatic mode; that is, the two most abundant ions in MS^1-2 ^were fragmented. The fragmentations were performed with 50% energy (0.85 V) with helium as the collision gas.

The anthocyanins in the samples were identified based on their UV-vis spectra (190-600 nm), mass spectra, and retention times relative to external standards, and comparison with literature reports on anthocyanins in chokeberries and blueberries [19,20].

The anthocyanins were quantified by external standard of cyanidin-3-glucoside (at 520 nm) in the concentration range 0.6-16.1 μg/mL, and the concentration of anthocyanins in the samples was expressed as μg/g caecal or colonic contents.

### HPLC-DAD-ESI-MSn analysis of phenolic metabolites

The HPLC apparatus, chromatographic column and MS conditions were the same as used for analysis of the anthocyanins, if not otherwise described. The separation of phenolic compounds was executed with mobile phases consisting of A; acetic acid/water (2/98, v/v) and B; acetic acid/acetonitrile (2/98, v/v) with the following gradient: 0-5 min 0% B, 5-44 min 0-25% B, 44-47 min 25-60% B, 47-56 min 60% B, 56-58 min 60-100% B, 58-61 min 100% B, 61-64 min 100-0% B. The column was allowed to equilibrate for 7 min between injections (10 μL). The column temperature was held at 30°C and the solvent flow rate was 0.25 mL/min.

The HPLC effluent was directed to the ESI interface, where the phenolic compounds were analysed in negative mode. The phenolic compounds in the samples were identified based on their UV-vis spectra (190-600 nm), mass spectra, and retention times relative to external standards, and comparison with literature reports about microbial metabolites of phenolic compounds [21-23].

The phenolic compounds were classified based on their characteristically UV-Vis spectra and quantified by external standards. Phenylacetic acid derivates i.e. 3,4-dihydroxyphenylacetic acid, 3-hydroxyphenylacetic acid and 4-hydroxyphenylacetic acid were quantified as 4-hydroxyphenylacetic acid at 280 nm. Phenylpropionic acid derivate i.e. 3-hydroxyphenylpropionic acid was quanitfied as 3-(4-hydroxyphenyl)propionic acid at 280 nm. Compounds with molecular mass same as 5-(3',4',5'-trihydroxyphenyl)-gamma-valerolactone and epicatechin were quantified as (-)-epicatechin. The concentrations of the polyphenolic compounds and metabolites were expressed as μg/g caecal or colonic content.

### Statistical analysis

All statistical analyses were performed in SigmaStat 3.1 (SPSS Inc., Chicago, Ill, USA). Differences between all groups were evaluated by Kruskal-Wallis one way ANOVA on ranks followed by all-pairwise-multiple-comparison Dunn's test. For evaluation of differences between two groups Mann-Whitney rank sum test was used. Results were considered statistically significant when p < 0.05. Values are presented as median (25^th^-75^th ^percentiles).

## Results

### Intestinal ischemia-reperfusion

All animals survived the I/R procedure, and reperfusion was performed and observed in all the animals. During the operation, colonic bleeding was observed in the following animals: 2 animals in B+LplH19-group, 3 animals in Ar-group, 1 animal in Ar+LplH19-group and 1 animal in LplH19-group, and anatomical abnormalities such as diminished and deformed organs were observed in one animal in I/R-control group. These, totally 8 animals were excluded from the data evaluation.

### Viable count

**Figure 1:**
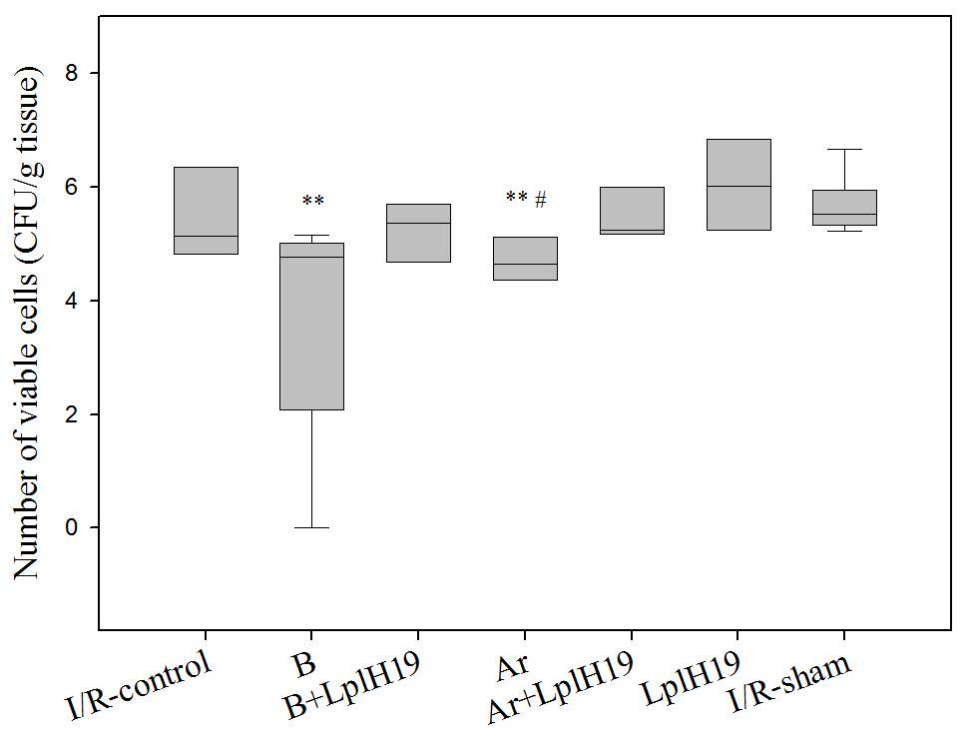
**Lactobacilli count of colonic mucosal tissue of mice after intestinal ischemia-reperfusion**. Viable count of lactobacilli of colonic mucosal tissue of mice administrated billberry (B), chokeberry (Ar) and/or strain *Lactobacillus plantarum *HEAL19 (LplH19). Lactobacilli count significantly decreased in Ar-group (**p = 0.008) and B-group (**p = 0.002) compared to LplH19-group. The count was higher in groups supplemented with LplH19 strain and significantly higher when LplH19 was added to chokeberry (Ar+LplH19-group) compared to only chokeberry-fed mice (Ar-group; #p = 0.029). Median values are indicated by the transverse line within the box and the top and bottom lines of each box represent the 25^th ^and 75^th ^percentiles.

The lactobacilli count of colonic tissue varied between 10^4 ^and 10^7 ^cfu/g. In B-group and Ar-group count of lactobacilli was significantly lower compared to LplH19-group (p = 0.002 and p = 0.008, respectively; Figure 1). The lactobacilli count was also significantly lower in Ar-group than in Ar+LplH19-group (p = 0.029; Figure 1). The count of lactobacilli was lower in B-group compared to the B+LplH19-group although the difference did not reach significance (p = 0.057).

The count of *Enterobacteriaceae *in colonic mucosal tissue was low and varied between <10^2 ^cfu/g and 10^4 ^cfu/g. No significant differences could be seen between the groups.

### Malondialdehyd (MDA)

**Figure 2:**
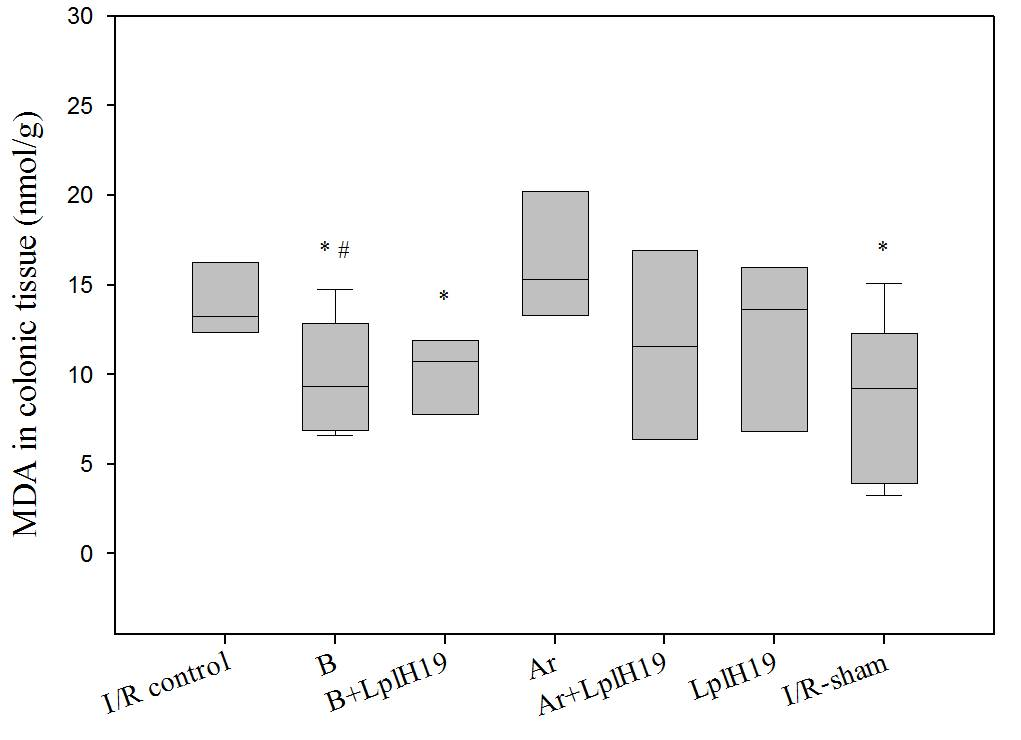
**Effects of experimental diets on lipid peroxidation in colon after intestinal ischemia-reperfusion**. The figure shows effects of billberry (B), chokeberry (Ar) and/or strain *Lactobacillus plantarum *HEAL19 (LplH19) administration on lipid peroxidation in colonic tissue. Significant decrease of lipid peroxidation (MDA) was observed in B-group (*p = 0.030), B+LplH19-group (*p = 0.021) and I/R-sham (*p = 0.014) compared to the I/R-control. A significantly lower MDA-value was also noted in B-group compared to Ar-group (#p = 0.016). Median values are indicated by the transverse line within the box and the top and bottom lines of each box represent the 25^th ^and 75^th ^percentiles.

In the colonic tissue, MDA was significantly lower in the Sham-group (p = 0.014) than in the I/R-control group. Significantly lower MDA was observed in B-group (p = 0.030) and B+LplH19-group (p = 0.021) compared to the I/R-control group. The B-group also showed a significantly lower MDA-value than the Ar-group (p = 0.016; Figure 2).

In the ceacal tissue, none of the dietary supplements significantly lowered the MDA-level.

### Anthocyanins

In the bilberry powder given to mice, 14 different anthocyanins were detected and identified; glucoside (glu), galactoside (gal) or arabinoside (arab) of cyanidin (Cy), delphinidin (Dp), petunidin (Pt), peonidin (Pn) and malvidin (Mv). Peonidin-3-arabinoside was not detected. Cy-3-gal and Dp-3-arab co-eluted in the chromatographic analysis. Glucosides, galactosides and arabinosides of Cy and Dp were the major anthocyanins in the native bilberry powder and contributed 67% to the total anthocyanin content. Pn-3-gal, Mv-3-arab, Pt-3-arab and Mv-3-gal were detected in the lowest concentrations and together represented 8% of anthocyanins in the powder.

In the chokeberry powder given to mice, four different anthocyanins were detected and identified i.e. Cy-3-gal (66%), Cy-3-glu (2%), Cy-3-arab (29%) and Cy-3-xyloside (xyl) (3%).

In the I/R-control, LplH19-group and sham-group no anthocyanins were detected in the caecal and colonic content (no supplement of berries to the diet). For the other treatment groups, large variations in anthocyanin composition pattern and in concentrations were observed between the different individuals within the same group.

In the colon of billberry-treated groups (B and B+LplH19) 12 different anthocyanins were identified (Table 1). Compared to the native bilberry powder, Pn-3-glu and Pn-3-gal were the only anthocyanins that were not detected. These two anthocyanins were absent in caecum as well. In caecum of the B-group and the B+LplH19-group, 9 and 8 different anthocyanins were identified, respectively. Compared to the colonic contents, Pt-3-gal, Mv-3-glu and Mv-3-gal were not recovered in the caecal contents of B-group (Table 1). In the caecum of B+LplH19-group, the same anthocyanins as in B-group were absent together with Pt-3-glu. As for the native bilberry powder, Cy-3-gal and Dp-3-arab could not be separated.

**Table 1:** Concentrations (μg/g) of anthocyanins in caecum and colon of mice administrated billberry (B) or chokeberry (Ar) alone or supplemented with strain *L. plantarum *HEAL19 (LplH19)

Comp.	B-group		B+LplH19-group		Ar-group		Ar+LplH19-group	
	caecum	colon	caecum	colon	caecum	colon	caecum	colon
**Dp-3-gal**	1.54	4.4*	0.96	2.58*	-	-	-	-
	(0.84-1.92)	(1.82-4.8)	(0.7-1.38)	(1.92-3.56)				
**Dp-3-glu**	1.04	2.7*	0.79	1.66*	-	-	-	-
	(0.72-1.22)	(1.28-3.21)	(0.52-0.96)	(1.3-2.4)				
**Dp-3-arab+**	2.94	9.44**	2.28	8.75**	-	-	-	-
**Cy-3-gal**	(2.23-3.35)	(7.64-12.54)	(1.8-3.05)	(6.5-12.78)				
**Cy-3-gal**	-	-	-	-	6.43	13.03**	5.09	12.27***
					(4.86-7.86)	(10.8-24.9)	(3.85-6.4)	(7.27-14.1)
**Cy-3-glu**	0.51	1.04*	0.46	0.72	-	-	-	-
	(0.47-0.63)	(0.7-1.18)	(0.44-0.48)	(0.56-1.17)				
**Cy-3-arab**	0.97	2.96**	0.8	1.61**	5.4^a^	12.16** ^b^	4.34^c^	9.97*** ^d^
	(0.75-1.1)	(1.83-3.16)	(0.58-0.96)	(1.3-1.84)	(3.96-6.24)	(9.1-23.34)	(2.65-5.4)	(6.41-12.8)
**Cy-3-xyl**	-	-	-	-	0.53	1.36**	0.48	2.06***
					(0.46-0.56)	(0.98-4.34)	(0.46-0.56)	(1.19-2.9)
**Pt-3-gal**	nd	1.12	nd	0.91	-	-	-	-
		(0.68-1.42)		(0.54-1.28)				
**Pt-3-glu**	0.75	1.34	nd	0.74*	-	-	-	-
	(0.7-0.8)	(0.74-1.47)		(0.71-1.16)				
**Pt-3-arab**	0.76	4.61**	0.6	3.04	-	-	-	-
	(0.68-1.03)	(2.33-8.0)	(0.52-0.68)	(1.38-8.9)				
**Mv-3-gal**	nd	0.85	nd	0.72	-	-	-	-
		(0.64-1.06)		(0.72-0.72)				
**Mv-3-glu**	nd	1.24	nd	1.00	-	-	-	-
		(1.03-46.0)		(0.61-31.75)				
**Mv-3-arab**	5.42	20.92*	4.38	16.76**	-	-	-	-
	(2.81-6.34)	(13.27-25.55)	(2.21-5.09)	(16.02-23.2)				

* denotes p < 0.05 compared to the caecum sample of the same treatment group; ** denotes p < 0.01 compared to the caecum sample of the same treatment group; *** denotes p < 0.001 compared to the caecum sample of the same treatment group; a denotes p < 0.001 compared to the caecum of B-group; b denotes p < 0.01 compared to the colon of B-group; c denotes p < 0.01 compared to the caecum of B+LplH19-group; d denotes p < 0.001 compared to the colon of B+LplH19-group; nd = not detected. Values are expressed as medians (25^th ^and 75^th ^percentiles).

The variety of different anthocyanins and the concentrations of the different anthocyanins were higher in the colon compared to the caecum. There was a trend that the concentration was higher in mice fed berries without addition of LplH19 (Table 1). Mv-3-arab was the major anthocyanin in both caecum and colon of the bilberry-groups. In both colon and caecum, arabinosides were detected in the higher amounts than glucosides and galactosides of the same anthocyanidin. In the bilberry powder galactosides and glucosides were detected in higher concentrations than arabinosides of the same anthocyanidin.

In both colon and caecum of chokeberry-treated groups 3 anthocyanins were identified i.e. Cy-3-gal, Cy-3-arab and Cy-3-xyloside (Table 1). Cy-3-glu that was present in the native chokeberry powder was not detected in the gut of mice. Concentration of anthocyanins was higher in the colon than in the caecum and also in this case there was a trend that the concentration was higher without addition of LplH19, except for Cy-3-xyl in colon (Table 1). Cy-3-gal was found in the highest and Cy-3-xyl in the lowest concentrations in both caecum and colon of the chokeberry-groups.

Cy-3-arab was found in both colon and caecum of both the bilberry and chokeberry groups. The concentration was higher in chokeberry treated animals. The concentration was especially high in colon.

### Phenolic metabolites

In animals not given berries in the diet, i.e. the I/R-control, the LplH19-group and the I/R-sham-group, no aromatic acids could be detected. Large individual differences in concentration and detectable levels of different phenolic compounds were observed.

Two metabolites with the same fragmentation pattern (*m/z *167→123) were detected at Rt 11.4 and 13.4 min (Table 2). On the basis of the MS fragmentation pattern and co-elution with authentic standard these compounds were identified as two isomers of 3,4-dihydroxyphenylacetic acid (diHPA). These compounds were detected in both caecum and colon of bilberry-fed groups.

**Table 2:** Aromatic acid metabolites detected in caecum and colon of mice administrated billberry (B) or chokeberry (Ar) alone or supplemented with strain *L. plantarum *HEAL19 (LplH19)

Rt (min)	Molecular mass	Parent ions ([M-H]^-^, *m/z*)	Product ions (*m/z*)	Compound	Detection site and group
**11.4**	168	167	123	3,4-Dihydroxyphenylacetic acid	Caecum and colon B and B+LplH19

**13.4**	168	167	123	3,4-Dihydroxyphenylacetic acid	Caecum and colon B and B+LplH19

**19.5**	224	223	123	Not identified I	Caecum and colon B and B+LplH19

**21.1**	224	223	123	Not identified II	Colon B and B+LplH19

**21.7**	224	223	123	Not identified III	Colon B and B+LplH19

**22.9**	152	151	107	4-Hydroxyphenylacetic acid	Caecum Ar and Ar+LplH19

**25.1**	152	151	107	3-Hydroxyphenylacetic acid	Colon Ar and Ar+LplH19

**27.4**	290	289	271	Non identified EC	Colon B and B+LplH19

**29**	191	190	146	Non identified	Caecum and colon B, B+LplH19, Ar,Ar+Lp19

**31.4**	166	165	121	3-Hydroxyphenylpropionic acid	Caecum and colon B and B+LplH19

Metabolites that appeared at Rt 19.5 minutes in caecum and colon of bilberry-fed groups and at Rt 21.1 and 21.7 min in colon of bilberry-fed groups showed the same fragmentation pattern, *m/z *223→123. A molecular mass of 224 Da corresponds to 5-(3',4,5'-trihydroxyphenyl)-gamma-valerolactone but the fragmentation pattern was not the same as previously shown for this compound (*m/z *223→179) [24].

In the caecal contents of chokeberry-fed groups, 4-hydroxyphenylacetic acid (4-HPA) was identified as metabolite eluted at Rt 22.9 min with fragmentation *m/z *151→107. A metabolite at Rt 25.1 min with the same fragmentation (*m/z *151→107) in colonic contents of chokeberry-fed groups was tentatively identified as 3-hydroxyphenylacetic acid (3-HPA) (Table 2).

In both ceacal and colonic contents of bilberry-fed groups, 3-hydroxyphenylpropionic acid (3-HPP) was identified at Rt 31.4 min (*m/z *165→121).

Two colonic metabolites occurring in the mice fed bilberries (B-group and B+LplH19-group) were not identified. One of the compounds had retention time at 27.4 min and a molecular mass of 290 Da, the same as epicatechin but the fragmentation pattern was different.

The other metabolite was eluted at 29 min and had molecular mass of 191 Da, which indicates the presence of nitrogen atoms. During fragmentation a loss of 44 amu (CO_2_) was observed (*m/z *190→146).

There were no significant differences in the concentrations of phenolic metabolites between caecum and colon or between the experimental groups (Table 3).

**Table 3:** Concentrations (μg/g) of aromatic acid metabolites in caecum and colon of mice administrated billberry (B) or chokeberry (Ar) alone or supplemented with strain *L. plantarum *HEAL19 (LplH19)

Compound	B-group		B+LplH19-group		Ar-group		Ar+LplH19-group	
	caecum	colon	caecum	colon	caecum	colon	caecum	colon
diHPA	73.3	70.1	79.2	66.0	nd	nd	nd	nd
isomer I	(68-101.6)	(55.2-96.8)	(57.3-87.7)	(41.5-87.9)				
diHPA	150.9	63	112.5	73.8	nd	nd	nd	nd
isomer II	(88.9-229.6)	(54-155.7)	(87.3-137.8)	(31.6-89.6)				
Ni I	29.9	34.2	22.7	33.8	nd	nd	nd	nd
	(27.2-45.8)	(33.7-44.3)	(22.7-22.7)	(33.8-33.8)				
Ni II	nd	19.3	nd	18.0	nd	nd	nd	nd
		(18.3-26.3)		(18.0-18.0)				
Ni III	nd	33.2	nd	31.4				
		(29.1-41.7)		(13.8-58.4)				
4-HPA	nd	nd	nd	nd	59.3	Nd	66.0	Nd
					(49.3-85.6)		(61.9-83.0)	
3-HPA	nd	nd	nd	nd	nd	42.6	nd	32.9
						(29.7-143.9)		(22.7-43.1)
Ni EC	nd	12.6	nd	11.5	nd	nd	nd	nd
		(10.6-17.1)		(9.3-19.1)				
3-HPP	310.2	124.6	258.4	159.1	nd	nd	nd	nd
	(209.7-360.1)	(84-141.6)	(129.9-340.9)	(128.8-186.9)				

nd = not detected, Ni = not identified. Values are expressed as medians (25^th ^and 75^th ^percentiles).

## Discussion

The gut microflora can play a crucial role in the nutritional and health status of the host via modulation of the immune and metabolic functions. The metabolic activity of gut microflora can transform dietary polyphenols to bioactive compounds with potential health effects. On the other hand, some polyphenols and their metabolites influence the growth and/or metabolic activity of the gut microflora and hence its composition and function. It has been shown that unabsorbed polyphenols and their metabolites selectively inhibit the growth of pathogens and stimulate growth of lactobacilli and bifidobacteria [25-27]. In the present study, we were not able to observe any changes in the count of the *Enterobacteriaceae *which is a family of gram negative bacteria with strong pro-inflammatory potential. *Enterobacteriaceae *were generally low in the colon of all tested animals. In previous studies [25-27] lactobacilli and bifidobacteria were either not affected or favoured by polyphenols. According to our results polyphenol-rich berries had a significantly lowering effect on lactobacilli since both bilberry and chokeberry supplementation without addition of LplH19 strain reduced the total count of lactobacilli on the colonic mucosa. Daily administration of LplH19 together with berries prevented the decrease of lactobacilli. Similar results were obtained in a study by Håkansson *et al *[28] where the total lactobacilli count on the caecal mucosa of rats fed blueberry husks were significantly lower compared to the group fed blueberry husk (*V. myrtillus*) with a probiotic mixture. An explanation may be that some species or strains of lactobacilli may be more susceptible to polyphenols than others. It has been shown that polyphenols reduced the adhesion ability of *L. rhamnosus *to intestinal epithelial cells [29]. LplH19 may be more resistant to polyphenols and/or possess higher metabolic activity towards bilberry and chokeberry polyphenols than the resident lactobacilli flora in the gut of the tested mice. Furthermore, addition of LplH19 may also have modified the colonic microflora including the *Lactobacillus *flora increasing the number of more polyphenol-resistant strains able to transform some of the phenolics. *L. plantarum *have a tannase activity and are able to degrade hydrolysable tannins [18]. Besides tannase, *L. plantarum *possess two inducible decarboxylases able to metabolise phenolic acids such as p-coumaric, ferulic and caffeic acids to their corresponding vinyl derivatives or into substituted phenyl propionic acids [30].

Oxidative stress induced by free radicals is an important cause of tissue injury recognized to occur in inflammation [31]. Intestinal ischemia-reperfusion injury, in which the SMA is occluded resulting in severe damage to epithelium, is a good model to study oxidative stress and hence inflammation in the gut [32]. Increased concentrations of malondialdehyde (MDA) indicate the degree of lipid peroxidation verifying the oxidative damage in tissue [3,5,7]. In this study MDA was also used as a marker for tissue injury. In accordance with other studies [3,5,7,28,33-35] MDA was significantly increased in I/R-control compared to the I/R-sham in colonic tissue. Groups supplemented with bilberry alone and also in combination with LAB-strain LplH19 significantly decreased MDA in the colon. The same reducing effect of bilberry on colonic MDA levels has been obtained in two studies where rats with dextrane sulphate sodium induced colitis were treated with blueberry husks (*V. myrtillus*) and probiotics [28,36]. Since lipid peroxidation is mediated by ROS, observed lowering effects of MDA by bilberries are probably due to their antioxidative and anti-inflammatory properties [9,37,38].

The caecal and colonic contents of mice fed chokeberry and bilberry had a dark purple to black colour suggesting high concentration of anthocyanins in the gut. Anthocyanin-profile of bilberries was relatively complex with 14 different anthocyanins identified in the powder. Of those anthocyanins 9 and 8 were found in the caecum of B-group and B+LplH19-group, respectively, and 12 in the colon. Pn-3-glu and Pn-3-gal were not found in either caecum or colon of mice fed bilberry. Study by Ichiyanagi *et al *[39] showed that malvidin and peonidin glucosides and galactosides were the major anthocyanins excreted in bile and urine of rats fed bilberry extract. The same compounds were also detected as the major anthocyanins in the liver and kidney. These results were confirmed in a study by Sakakibara *et al *[40]. In the present study, peonidin glycosides may have been absorbed before they reached the caecum or degraded by caecal microflora and for that reason could not be detected.

It has been shown that bioavailability was higher for more hydrophobic aglycones such as Mv, Pn and Pt than for Dp and Cy [39,40]. In the present study, in addition to Pn glycosides also Mv-3-gal, Mv-3-glu, Pt-3-gal and Pt-3-glu were missing in the caecum of B-group and B+LplH19-group compared to the bilberry powder and also compared to the colon. The results of the present study suggest that galactosides and glucosides of Mv, Pn and Pt may have been absorbed to some extent from the upper part of the GIT and to certain degree metabolised by microflora in the caecum. The non-absorbed fraction reached the colon. Considering the fact that the concentration of anthocyanins in our study was significantly higher in the colon compared to the caecum it seems that anthocyanins were poorly absorbed and eventually reached the colon where they were accumulated [41]. Since anthocyanins have been reported to have antioxidative and anti-inflammatory properties [42] their accumulation in the colon may have a direct impact on the gut mucosa, including protection against oxidative stress. Especially the concentration of arabinosides was high compared to the gals and glus of the same aglycone. Arab, seemed to be more stable in the gut than gal and glu. This has been confirmed by other authors [39,43] and it was suggested that arab, as a pentoside, may be more resistant to microbial degradation.

Keppler *et al *[44] found that mono- and diglucosides of Cy, Mv and Pn were degraded by microflora to vanillic, syringic, gallic and protocatechuic acids in an *in vitro *model. None of the mentioned metabolites was detected in our study.

Anthocyanins were analysed in the berry powders but not in the experimental diets (standard chow supplemented with berry powder) given to the mice. The lack of the anthocyanin-concentrations in the experimental diets makes it difficult to know exactly how much of the consumed anthocyanins that was absorbed through the GIT.

Fruit juice from *Aronia melanocarpa *significantly decreased lipid peroxidation (MDA) in liver and plasma of rats with CCl_4_-induced acute liver damage and indomethacin-induced gastric mucosal damage [33,34]. The ability of anthocyanins in chokeberries to scavenge free radicals was suggested to play the main role on the reduction of MDA. In the present study, MDA levels were not significantly decreased in chokeberry groups and LplH19-group. The difference in the results may depend on the form of chokeberry used in the studies. In our study we used another species of chokeberry, namely *Aronia × prunifolia *instead of *Aronia melanocarpa *(black chokeberry). *A. prunifolia*, a purple chokeberry, is a hybrid between black chokeberry and red chokeberry (*A. arbutifolia*) [45]. Further, factors such as cultivar, fertilization, maturation of the berries, harvest date and habitat/location may exert a differential influence on quantity and profile of phenolics in *Aronia *fruits [46] and hence antioxidant properties. Further, animals in the present study had a free access to feed consisting of standard chow mixed with chokeberry powder from whole, freeze-dried berries while above mentioned authors used chokeberry juice administrated by direct stomach intubation. The possible differences in the microflora between animals (rats versus mice) used in the studies may also have an influence on the results.

In the present study four anthocyanins were detected in the chokeberry powder and three of these anthocyanins were found in the caecum and colon of mice. The main anthocyanin in the chokeberry was Cy-3-gal (66%). It was also found in the highest concentration in the caecum and colon of mice. The concentration of Cy-3-arab was only slightly lower compared to the Cy-3-gal in the intestines, although concentration of Cy-3-gal was more than 30% higher in the chokeberry powder compared to Cy-3-arab. This indicates that Cy-3-gal is to higher extent absorbed and/or degraded by gut microflora than Cy-3-arab. In the chokeberry powder, 3% of anthocyanins was Cy-3-xyl. Almost the same percentage of Cy-3-xyl was found in the caecum and colon of mice which indicates that Cy-3-xyl was quite resistant to the degradation in the gut. Arabinoside and xyloside seem to be more stabile in the gut compared to the sugar hexoses. This is in agreement with the results obtained by He *et al *[43].

Only 2% of the anthocyanins found in the chokeberry powder was Cy-3-glu and it was not detected in the intestines. Matuschek *et al *[47] showed that Cy-3-glu was mainly absorbed from jejunum. Tsuda *et al *[48] observed that Cy-3-glu but also its degradation product protocatechuic acid (PCA) [44] were detected in the jejunum and plasma of rats fed Cy-3-glu. In chokeberry fed mice, Cy-3-glu and its possible degradation product PCA, were probably absorbed from the small intestine and for that reason could not be detected in the caecum or colon.

There was a trend that the concentrations of the identified anthocyanins in the study were lower in both colon and caecum of mice fed either bilberry or chokeberry with addition of LplH19. These results indicate that supplementation with the *Lactobacillus *strain modified the composition of colonic microflora, probably increasing the number of microbes with metabolic activity towards higher degradation of anthocyanins, or Lp1H19 by itself to a certain degree can degrade anthocyanins.

In agreement with other studies, different aromatic acids were detected in the caecum and colon of mice fed polyphenol-rich diets [12,21,49,50]. Detected phenolic acids are probably metabolites of microbial degradation of different polyphenols present in the berries that were not absorbed from small intestine or excreted in the bile.

3,4-dihydroxyphenylacetic acid (diHPA) was identified in bilberry-fed groups. This metabolite has been found in other studies and was suggested as the main product of proanthocyanidin (dimer) microbial degradation [12,21,49,51] but also quercetin degradation [52]. diHPA was identified in freeze-dried blueberries after inoculation with faeces from two human volunteers [37]. Since both procyanidins (epicatechin-monomers linked with A-type and B-type bonds) and quercetin glycosides are present in the bilberries [20,53] diHPA in the present study most probably resulted from degradation of these compounds.

Although procyanidins are identified as the major class of polyphenols in chokeberries and flavonols, mainly quercetin glycosides, represent 1.3% of chokeberry phenolics, we did not detect diHPA in the chokeberry-fed mice [46]. Instead we detected 3-hydroxyphenylacetic and 4-hydroxyphenylacetic acids which are suggested to be metabolites of diHPA dehydroxylation at meta and para positions [49,51]. Rios *et al *[49] described diHPA as an intermediate between flavanols and the more dehydroxylated 3-HPA and 4-HPA. It has been shown that diHPA exhibit a considerable antiproliferative activity in LNCaP prostate cancer and in HCT116 colon cancer cells [54]. It also had more potent anti-platelet aggregation activity compared to its parent compound quercetin and showed more potent cytotoxicity against tumor cell lines than 4-HPA [55]. diHPA exerted anti-inflammatory properties by reducing the secretion of pro-inflammatory cytokines, TNF-α, IL-1β and IL-6, involved in the early stages of atherosclerosis from lipopolysaccharide (LPS)-stimulated human peripheral blood mononuclear cells (PBMC). Monohydroxylated acids, such as 3-HPA, did not produce the significant changes in cytokine secretion [56].

3-hydroxyphenylpropionic acid (3-HPP) was another metabolite identified in the colon and caecum of bilberry fed mice. Several compounds have been proposed as precursors of 3-HPP. Rios *et al *[49] detected 3-HPP in the human urine after consumption of flavanol-rich chocolate containing monomers of epicatechin, catechin and oligomers up to decamers. Even other authors found 3-HPP after microbial degradation of procyanidins (monomers and dimers) [12,21,51]. Another suggested source of 3-HPP is microbial degradation of chlorogenic acid [57]. Especially bilberries, but also chokeberries are rich sources of chlorogenic acid [9,46], however 3-HPP was detected only in bilberry treated groups.

Metabolites found in the caecum and colon of bilberry treated mice, but not in chokeberry-groups, that had the same molecular weight as phenylvalerolactone but different fragmentation pattern, may also be degradation products of epicatechin or procyanidins of lower polymerization degree, as suggested by different studies [24,51,58]. According to Gonthier *et al *[21] the yield of microbial metabolites decreases as the degree of procyanidin polymerization increases due to reduced accessibility of the substrate or interaction of procyanidin with proteins in the gut lumen. More than 80% of procyanidins in chokeberries have higher polymerization degree than 10-mers and only around 4% are monomers, dimers and trimers [46]. The higher content of procyanidin polymers in chokerberry compared to the bilberry may be the reason that fewer metabolites were detected in the caecum and colon of chokeberry treated groups. Aromatic acid metabolites have reducing and antioxidant properties and since they are more easily absorbed from the colon into the circulation they may significantly contribute to the protection against oxidative stress not only locally in the intestines but also in other tissues.

## Conclusions

Oxidative stress injury caused by ischemia-reperfusion in mice was significantly suppressed by administration of bilberry alone and together with probiotic strain *L. plantarum *HEAL19. Chokeberry supplementation did not have antioxidative effects in the present study, although Zheng *et al *[9] showed that chokeberry possesses higher antioxidative capacity *in vitro *than bilberry. Bilberries have the lower content of highly polymerized procyanidins than chokeberries which make them more accessible for microbial degradation and hence more antioxidative metabolites are produced in the gut of bilberry-fed mice. Compared to chokeberries, bilberries also have more complex composition of different anthocyanins that accumulate in the colon. All those factors may be responsible for the antioxidative effects of bilberries *in vivo*. The variation of anthocyanins and microbial metabolites was especially large in the colon of bilberry treated groups where the antioxidative effect was evident. Bilberry in combination with *L. plantarum *HEAL19 strain was as efficient as bilberry alone in suppressing the oxidative stress in the intestines but supplementation with *L. plantarum *HEAL19 seemed to influence the intestinal microflora towards more polyphenol-tolerant and metabolically active microbiota. More inflammatory markers beside MDA should be tested to confirm the anti-inflammatory effects of bilberries and probiotics in an oxidative stress induced intestinal inflammation.

## Abbreviations used

GIT: gastrointestinal tract; CFU: colony forming units; DAD: diode array absorbance detector; ESI: electrospray ionization; HPLC: high-performance liquid-chromatography; MS: mass spectroscopy; UV-vis: ultraviolet-visible light; Rt: retention time

## Competing interests

The authors declare that they have no competing interests.

## Authors' contributions

MJ has contributed to conception and design of the study, analysis and interpretation of data and mainly drafting the manuscript. KA has contributed to analysis of polyphenols and interpretation of HPLC-DAD-ESI-MS data and has been involved in drafting and revising the manuscript critically. GIB helped with interpretation of HPLC-DAD-ESI-MS data and has been involved in revising the manuscript critically. BJ has been involved in interpretation of data and revised the manuscript critically. GM and SA participated in conception and design, interpretation of data and have been involved in revising the manuscript critically. All authors read and approved the final manuscript.

## Pre-publication history

The pre-publication history for this paper can be accessed here:

http://www.biomedcentral.com/1472-6882/11/8/prepub

## References

[B1] Arumugam TV Shiels AA Woodruff TM Granger DN Taylor AM The role of the complement system in ischemia-reperfusion injury Shock 2004 21 401 409 10.1097/00024382-200405000-00002 15087815

[B2] Collard CD Gelman S Pathophysiology, clinical manifestations, and prevention of ischemia-reperfusion injury Anesthesiology 2001 94 1133 38 10.1097/00000542-200106000-00030 11465607

[B3] Teke Z Kabay B Aytekin FO Yenisey C Demirkan NC Sacar M Erdem E Oden A Pyrrolidine dithiocarbamate prevents 60 minutes of warm mesenteric ischemia/reperfusion injury in rats Am J Surg 2007 194 255 262 10.1016/j.amjsurg.2006.06.054 17618816

[B4] Mallick IH Yang W Winslet MC Seifalian AM Ischemia-reperfusion injury of the intestine and protective strategies against injury Dig Dis Sci 2004 49 1359 1377 10.1023/B:DDAS.0000042232.98927.91 15481305

[B5] Ozkan OV Yuzbasioglu MF Ciralik H Kurutas EB Yonden Z Aydin M Bulbuloglu E Semerci E Goksu M Atli Y Bakan V Duran N Resveratrol, a natural antioxidant, attenuates intestinal ischemia/reperfusion injury in rats Tahoku J Exp Med 2009 218 251 258 10.1620/tjem.218.251 19561396

[B6] Mojzis J Hviscová K Germanova D Bukovicová D Mirossay L Protective effect of quercetin on ischemia/reperfusion-induced gastric mucosal injury in rats Physiol Res 2001 50 501 506 11702854

[B7] Muiá C Mazzon E Di Paola R Genovese T Menegazzi M Caputi AP Suzuki H Cuzzocrea S Green tea polyphenol extract attenuates ischemia/reperfusion injury of the gut Naunyn-Schmiedeberg's Arch Pharmacol 2005 371 364 374 10.1007/s00210-005-1076-0 15997392

[B8] Gao X Björk L Trajkovsli V Uggla M Evaluation of antioxidant activities of rosehip ethanol extracts in different test systems J Sci Food Agric 2000 80 2021 2027 10.1002/1097-0010(200011)80:14<2021::AID-JSFA745>3.0.CO;2-2

[B9] Zheng W Wang SY Oxygen radical absorbing capacity of phenolics in blueberries, cranberries, chokeberries and lingonberries J Agric Food Chem 2003 51 502 509 10.1021/jf020728u 12517117

[B10] Prior RL Cao G Martin A Sofic E McEwen J O'Brien C Lischner N Ehlenfeldt M Kalt W Krewer G Mainland CM Antioxidant capacity as influenced by total phenolic and anthocyanin content, maturity, and variety of *Vaccinium *species J Agric Food Chem 1998 46 2686 2693 10.1021/jf980145d

[B11] Bermúdez-Soto M-J Tomás-Barberán F-A García-Conesa M-T Stability of polyphenols in chokeberry (*Aronia melanocarpa*) subjected to in vitro gastric and pancreatic digestion Food Chem 2007 102 865 874

[B12] Gonthier M-P Cheynier V Donovan LJ Manach C Morand C Mila I Lapierre C Rémésy C Scalbert A Microbial aromatic acid metabolities formed in the gut account for a major fraction of the polyphenols excreted in urine of rats fed red wine polyphenols J Nutr 2003 133 461 467 12566484 10.1093/jn/133.2.461

[B13] Manach C Scalbert A Morand C Rémésy C Jiménez L Polyphenols: food sources and bioavailability^1,2^ Am J Clin Nutr 2004 79 727 747 15113710 10.1093/ajcn/79.5.727

[B14] Jenner AM Rafter J Halliwell B Human fecal water content of phenolics: the extent of colonic exposure to aromatic compounds Free Radic Biol Med 2005 38 763 772 10.1016/j.freeradbiomed.2004.11.020 15721987

[B15] Lappara JM Sanz Y Interactions of gut microbiota with functional food components and nutraceuticals Pharmacol Res 2010 61 219 225 10.1016/j.phrs.2009.11.001 19914380

[B16] Davis CD Milner JA Gastrointestinal microflora, food components and colon cancer prevention J Nutr Biochem 2009 20 743 752 10.1016/j.jnutbio.2009.06.001 19716282 PMC2743755

[B17] Isolauri E Kirjavainen PV Salminen S Probiotics: a role in treatment of intestinal infection and inflammation? Gut 2002 50 Suppl III iii54 iii59 10.1136/gut.50.suppl_3.iii54 11953334 PMC1867676

[B18] Osawa R Kuroiso K Goto S Shimizu A Isolation of tannin-degrading lactobacilli from humans and fermented foods Appl Environ Microbiol 2000 66 3093 3097 10.1128/AEM.66.7.3093-3097.2000 10877812 PMC92117

[B19] Wu X Gu L Prior RL McKay S Characterization of anthocyanins and proanthocyanidins in some cultivars of *Ribes*, *Aronia*, and *Sambucus *and their antioxidant capacity J Agric Food Chem 2004 52 7846 7856 10.1021/jf0486850 15612766

[B20] Prior RL Lazarus SA Cao G Muccitelli H Hammerstone JF Identification of procyanidins and anthocyanidins in blueberries (*Vaccinium Spp*.) using high-performance liquid chromatography/mass spectrometry J Agric Food Chem 2001 49 1270 1276 10.1021/jf001211q 11312849

[B21] Gonthier M-P Donovan LJ Texier O Felgines C Rémésy C Scalbert A Metabolism of dietary procyanidins in rats Free Radic Biol Med 2003 35 837 844 10.1016/S0891-5849(03)00394-0 14556848

[B22] Gonthier M-P Rios LY Verny M-A Rémésy C Scalbert A Novel liquid chromatography-electrospray ionization mass spectrometry method for the quantification in human urine of microbial aromatic acid metabolites derived from dietary polyphenols J Chromatogr B 2003 789 247 255 10.1016/S1570-0232(03)00073-4 12742116

[B23] Urpi-Sarda M Monagas M Khan N Llorach R Lamuela-Raventόs RM Jáuregui O Estruch R Izquierdo-Pulido M Andrés-Lacueva C Targeted metabolic profiling of phenolics in urine and plasma after regular consumption of cocoa by liquid chromatography-tandem mass spectrometry J Chromatogr A 2009 1216 7258 7267 10.1016/j.chroma.2009.07.058 19671472

[B24] Meng X Sang S Zhu N Lu H Sheng S Lee M-J Ho C-T Yang CS Identification and characterization of methylated and ring-fission metabolites of tea catechins formed in humans, mice and rats Chem Res Toxicol 2002 15 1042 1050 10.1021/tx010184a 12184788

[B25] Lee CH Jenner AM Low SC Lee YK Effect of tea phenolics and their aromatic fecal bacterial metabolites on intestinal microbiota Res Microbiol 2006 157 876 884 10.1016/j.resmic.2006.07.004 16962743

[B26] Tzounis X Vulevic J Kuhnle GGC George T Leonczak J Gibson GR Kwik-Uribe C Spencer JPE Flavonol monomer-induced changes to the human faecal microflora Br J Nutr 2008 99 782 792 10.1017/S0007114507853384 17977475

[B27] Puupponen-Pimiä R Nohynek L Hartmann-Schmidlin S Kähkönen M Heinonen M Määttä-Riihinen K Oksman-Caldentey K-M Berry phenolics selectively inhibit the growth of intestinal pathogens J Appl Microbiol 2005 98 991 1000 15752346 10.1111/j.1365-2672.2005.02547.x

[B28] Håkansson Å Bränning C Adawi D Molin G Nyman M Jeppsson B Ahrné S Blueberry husks, rye bran and multi-strain probiotics affect the severity of colitis induced by dextran sulphate sodium Scand J Gastroenterol 2009 44 1213 1225 19670079 10.1080/00365520903171268

[B29] Parkar SG Stevenson DE Skinner MA The potential influence of fruit polyphenols on colonic microflora and human gut health Int J Food Microbiol 2008 124 295 298 10.1016/j.ijfoodmicro.2008.03.017 18456359

[B30] Barthelmebs L Divies C Cavin J-F Knockout of the p-coumarate dexarboxylase gene from *Lactobacillus plantarum *reveals the existence of two other inducible enzymatic activities involved in phenolic acid metabolism App Environ Microbiol 2000 66 3368 3375 10.1128/AEM.66.8.3368-3375.2000 PMC92157 10919793

[B31] Thomson A Hemphill D Jeejeebhoy KN Oxidative stress and antioxidants in intestinal disease Dig Dis 1998 16 152 58 10.1159/000016859 9618134

[B32] Stallion A Kou TD Latifi SQ Miller KA Dahms BB Dudgeon DL Levine AD Ischemia/reperfusion: a clinically relevant model of intestinal injury yielding systemic inflammation J Pediatr Surg 2005 40 470 477 10.1016/j.jpedsurg.2004.11.045 15793720

[B33] Valcheva-Kuzmanova S Borisova P Galunska P Krasnaliev I Belcheva A Hepatoproctective effect of the natural fruit juice from *Aronia melanocarpa *on carbon tetra-chloride-induced acute liver damage in rats Exp Toxicol Pathol 2004 56 195 201 10.1016/j.etp.2004.04.012 15625789

[B34] Valcheva-Kuzmanova S Marazova K Krasnaliev I Galunska P Borisova P Belcheva A Effect of *Aronia melanocarpa *fruit juice on indomethacin-induced gastritic mucosal damage and oxidative stress in rats Exp Toxicol Pathol 2005 56 385 392 10.1016/j.etp.2005.01.001 15945278

[B35] Jakesevic M Håkansson Å Adawi D Jeppsson B Rumpunen K Ekholm A Ahrné S Molin G Antioxidative protection of dietary rosehips and polyphenol active lactobacilli in mice subjected to intestinal oxidative stress by ischemia-reperfusion Microb Ecol Health Dis 2009 21 193 202 10.3109/08910600903429045

[B36] Osman N Adawi D Ahrné S Jeppsson B Molin G Probiotics and blueberry attenuate the severity of dextran sulfate sodium (DSS)-induced colitis Dig Dis Sci 2008 53 2464 2473 10.1007/s10620-007-0174-x 18274903

[B37] Russell WR Labat A Scobbie L Duncan S Availibility of blueberry phenolics for microbial metabolism in the colon and the potential inflammatory implications Mol Nutr Food Res 2007 51 726 731 10.1002/mnfr.200700022 17487929

[B38] Kähkönen MP Heinämäki J Ollilainen V Heinonen M Berry anthocyanins: isolation, identification and antioxidant activities J Sci Food Agric 2003 83 1403 1411

[B39] Ichiyanagi T Shida Y Rahman MM Hatano Y Konishi T Bioavailability and tissue distribution of anthocyanins in bilberry (*Vaccinium Myrtillus *L.) extract in rats J Agric Food Chem 2006 54 6578 6587 10.1021/jf0602370 16939312

[B40] Sakakibara H Ogawa T Koyanagi A Kobayashi S Goda T Kumazawa S Kobayashi H Shimoi K Distribution and excretion of bilberry anthocyanins in mice J Agric Food Chem 2009 57 7681 7686 10.1021/jf901341b 19663426

[B41] Kahle K Kraus M Scheppach Ackermann M Ridder F Richling E Studies on apple and blueberry fruit constituents: Do the polyphenols reach the colon after ingestion? Mol Nutr Food Res 2006 50 418 423 10.1002/mnfr.200500211 16548015

[B42] Wang H Nair MG Strasburg GM Chang Y-C Booren AM Gray JI DeWitt DL Antioxidant and anti-inflammatory activities of anthocyanins and their aglycon, cyaniding, from tart cherries J Nat Prod 1999 62 294 296 10.1021/np980501m 10075763

[B43] He J Magnuson BA Giusti MM Analysis of anthocyanins in rat intestinal contents-impact of anthocyanin chemical structure on fecal excretion J Agric Food Chem 2005 53 2859 2866 10.1021/jf0479923 15826031

[B44] Keppler K Humpf H-U Metabolism of anthocyanins and their phenolic degradation products by the intestinal microflora Bioorg Med Chem 2005 13 5195 5205 10.1016/j.bmc.2005.05.003 15963727

[B45] Kokotkiewicz A Jaremicz Z Luczkiewicz M *Aronia *plants: a review of traditional use, biological activities, and perspectives for modern medicine J Med Food 2010 13 255 269 10.1089/jmf.2009.0062 20170359

[B46] Kulling SE Rawel HM Chokeberry (*Aronia melanocarpa*) - a review on the characteristic components and potential health effects Planta Med 2008 74 1625 1634 10.1055/s-0028-1088306 18937167

[B47] Matuschek MC Hendriks WH McGhie TK Reynolds GW The jejunum is the main site of absorption for anthocyanins in mice J Nutr Biochem 2006 17 31 36 10.1016/j.jnutbio.2005.04.005 16098729

[B48] Tsuda T Horio F Osawa T Absorption and metabolism of cyaniding-3-O-β-D-glucoside in rats FEBS letters 1999 449 179 182 10.1016/S0014-5793(99)00407-X 10338127

[B49] Rios LY Gonthier MP Rémésy C Mila I Lapierre C Lazarus SA Williamson G Scalbert A Chocolate intake increases urinary excretion of polyphenol-derived phenolic acids in healthy human subjects Am J Clin Nutr 2003 77 912 918 12663291 10.1093/ajcn/77.4.912

[B50] Wu X Pittman HE III Hager T Hager A Howard L Prior RL Phenolic acids in black raspberry and in the gastrointestinal tract of pigs following ingestion of black raspberry Mol Nutr Food Res 2009 53 S76 S84 10.1002/mnfr.200800231 19199287

[B51] Appeldoorn MM Vincken J-P Aura A-M Hollman PCH Gruppen H Procyanidin dimers are metabolized by human microbiota with 2-(3,4-dihydroxyphenyl)acetic acid and 5-(3,4-dihydroxyphenyl)-γ-velorelactone as the major metabolites J Agric Food Chem 2009 57 1084 1092 10.1021/jf803059z 19191673

[B52] Aura A-M O'Leary KA Williamson G Ojala M Bailey M Puupponen-Pimiä R Nuutila AM Oksman-Caldentey K-M Poutanen K Quercetin derivates are deconjugated and converted to hydroxyphenylacetic acids but not methylated by human fecal flora in vitro J Agric Food Chem 2002 50 1725 1730 10.1021/jf0108056 11879065

[B53] Määttä-Riihinen KR Kamal-Eldin A Mattila PH González-Paramás AM Törrönen A-R Distribution and contents of phenolic compounds in eighteen Scandinavian berry species J Agric Food Chem 2004 52 4477 4486 15237955 10.1021/jf049595y

[B54] Gao K Xu A Krul C Venema K Liu Y Niu Y Lu J Bensoussan L Seeram NP Heber D Henning SM Of the major phenolic acids formed during human microbial fermentation of tea, citrus and soy flavonoid supplements, only 3,4-dihydroxyphenylacetic acid has proliferative activity J Nutr 2006 136 52 57 16365058 10.1093/jn/136.1.52

[B55] Kim D-H Kim S-Y Park S-Y Han MJ Metabolism of quercitrin by human intestinal bacteria and its relation to some biological activities Bio Pharm Bull 1999 22 749 751 10.1248/bpb.22.749 10443478

[B56] Monagas M Khan N Andrés-Lacueva C Urpí-Sardá M Vázquez-Agell M Lamuela-Raventόs RM Estruch R Dihydroxylated phenolic acids derived from microbial metabolism reduce lipopolysaccharide-stimulated cytokine secretion by human peripheral blood mononuclear cells Br J Nutr 2009 102 201 206 10.1017/S0007114508162110 19586571

[B57] Gonthier M-P Verny M-A Besson C Rémésy C Scalbert A Chlorogenic acid bioavailability largely depends on its metabolism by the microflora in rats J Nutr 2003 133 1853 1859 12771329 10.1093/jn/133.6.1853

[B58] Selma MV Espín JC Tomás-Barberán FA Interaction between phenolics and gut microbiota: role in human health J Agric Food Chem 2009 57 6485 6501 10.1021/jf902107d 19580283

